# Faecal Microbiota transplantation affects liver DNA methylation in Non-alcoholic fatty liver disease: a multi-omics approach

**DOI:** 10.1080/19490976.2023.2223330

**Published:** 2023-06-14

**Authors:** Daniela Stols-Gonçalves, Anne Linde Mak, Mette S. Madsen, Eduard W. J. van der Vossen, Eveline Bruinstroop, Peter Henneman, Femke Mol, Torsten P. M. Scheithauer, Loek Smits, Julia Witjes, Abraham Stijn Meijnikman, Joanne Verheij, Max Nieuwdorp, Adriaan G. Holleboom, Evgeni Levin

**Affiliations:** a Department of Vascular Medicine, Amsterdam University Medical Centre, Amsterdam, The Netherlands; b Amsterdam Gastroenterology Endocrinology Metabolism (AGEM) Institute, Amsterdam UMC, University of Amsterdam, Amsterdam, The Netherlands; c Gubra, Hørsholm, Denmark; d Novo Nordisk Foundation Center for Biosustainability, Technical University of Denmark, Kongens Lyngby, Denmark; e Department of Endocrinology, Amsterdam University Medical Centre, Amsterdam, The Netherlands; f Department of Human Genetics, Amsterdam University Medical Centre, Amsterdam, The Netherlands; g Horaizon BV, Delft, The Netherlands; h Department of Internal Medicine, Amsterdam University Medical Centre (UMC), Vrije Universiteit (VU) University Medical Centre, Amsterdam, Netherlands; i Department of Pathology, Amsterdam University Medical Centre, Amsterdam, The Netherlands

**Keywords:** DNA methylation, faecal microbiota transplantation (FMT), non-alcoholic fatty liver disease (NAFLD), multi-omics analysis

## Abstract

Individuals with nonalcoholic fatty liver disease (NAFLD) have an altered gut microbiota composition. Moreover, hepatic DNA methylation may be altered in the state of NAFLD. Using a fecal microbiota transplantation (FMT) intervention, we aimed to investigate whether a change in gut microbiota composition relates to altered liver DNA methylation in NAFLD. Moreover, we assessed whether plasma metabolite profiles altered by FMT relate to changes in liver DNA methylation. Twenty-one individuals with NAFLD underwent three 8-weekly vegan allogenic donor (*n* = 10) or autologous (*n* = 11) FMTs. We obtained hepatic DNA methylation profiles from paired liver biopsies of study participants before and after FMTs. We applied a multi-omics machine learning approach to identify changes in the gut microbiome, peripheral blood metabolome and liver DNA methylome, and analyzed cross-omics correlations. Vegan allogenic donor FMT compared to autologous FMT induced distinct differential changes in I) gut microbiota profiles, including increased abundance of *Eubacterium siraeum* and potential probiotic *Blautia wexlerae*; II) plasma metabolites, including altered levels of phenylacetylcarnitine (PAC) and phenylacetylglutamine (PAG) both from gut-derived phenylacetic acid, and of several choline-derived long-chain acylcholines; and III) hepatic DNA methylation profiles, most importantly in Threonyl-TRNA Synthetase 1 (*TARS)* and Zinc finger protein 57 (*ZFP57)*. Multi-omics analysis showed that *Gemmiger formicillis* and *Firmicutes bacterium*_*CAG*_170 positively correlated with both PAC and PAG. *E siraeum* negatively correlated with DNA methylation of cg16885113 in *ZFP57*. Alterations in gut microbiota composition by FMT caused widespread changes in plasma metabolites (e.g. PAC, PAG, and choline-derived metabolites) and liver DNA methylation profiles in individuals with NAFLD. These results indicate that FMTs might induce metaorganismal pathway changes, from the gut bacteria to the liver.

## Introduction

Non-alcoholic fatty liver disease (NAFLD) is a spectrum of liver disease ranging from isolated steatosis to steatohepatitis (NASH), which can eventually progress to NASH-fibrosis, cirrhosis and hepatocellular carcinoma.^1^ NAFLD is estimated to affect 25% of the global population. The prevalence of NAFLD rises proportionally with an increase in body mass index (BMI), reaching over 90% in individuals with a BMI of ≥30 kg/m^2^.^2^ Moreover, 55% of individuals with type 2 diabetes mellitus (T2DM) have NAFLD.^3–5^ NAFLD is thus regarded as the hepatic component of the metabolic syndrome.^6^ Individuals with NAFLD are at an increased risk of death, with hepatic fibrosis as the strongest predictor of mortality. There is also a higher incidence of cardiovascular events in individuals with NAFLD.^7,8^ Despite this considerable disease burden, to date, no proven or registered pharmacotherapy is available to reduce the burden of NAFLD.

The gut microbiome is emerging as an interesting therapeutic target for metabolic diseases. Gut microbiota composition can be modulated by fecal microbiota transplantation (FMT),^9^ a procedure in which the feces of a healthy individual is transplanted into the gastrointestinal tract of a recipient with the aim of restoring a healthy balance of gut bacteria. FMT, potentially delivered through oral capsules, could become a useful therapeutic strategy for the management of metabolic diseases.^10^

Indeed, changes in gut microbiota composition likely have effects beyond the intestine.^11^ Recent data suggest that gut microbiota and gut microbiota derived metabolites correlate with epigenetic modifications. This is particularly known for DNA methylation, an epigenetic modification that has been associated with several metabolic diseases, including obesity and T2DM.^12,13^ A recent study comparing germ-free and conventionally raised mice demonstrated that exposure to commensal microbiota significantly increased DNA methylation at regulatory elements of intestinal genes.^14^ Moreover, it has been demonstrated in mice fed a high-fat diet that an alteration of gut microbiota induced by antibiotics was associated with changes in DNA methylation in epididymal fat. In this study, the authors observed reduced DNA methylation in the promoter region of the adiponectin and resistin genes as well as downregulated expression of DNA methyltransferases 1 (DNMT1) and DNA methyltransferases 3a (DNMT3a).^15^

The correlation between gut microbiota and DNA methylation in metabolic diseases has also been shown in humans. In a study on the link between the gut microbiota and global DNA methylation profiles in obesity, study participants were separated into two groups based on their Bacteroidetes-to-Firmicutes ratio (high or low). Individuals in these two groups had distinct global DNA methylation patterns. Differences in methylation of genes involved in glucose and energy homeostasis were accompanied by different levels of expression, e.g. in Histone deacetylase 7 gene (*HDAC7*) and Insulin – like growth factor protein gene (*IGF2BP2*).^16^ Guo *et al*. have demonstrated that obesity-prone individuals exhibit diabetes-related DNA methylation signatures despite being normal in weight and BMI. Moreover, they showed that these diabetes-related DNA methylation features are transferable through the gut microbiota, by performing an FMT from obesity-prone human donors to mice.^17,18^ Our group has previously shown that lean donor FMT affects the gut microbiome and the DNA methylome of peripheral blood mononuclear cells in individuals with the metabolic syndrome. More specifically, the introduction of Prevotella species after allogenic FMT correlated with methylation of Actin filament-associated protein 1 (*AFAP*), a gene involved in mitochondrial function.^11^

There are a few studies that have reported on DNA methylation specifically in NAFLD. Both nuclear and mitochondrial DNA methylation have been implicated in the pathogenesis of NAFLD.^19,20^ A myriad of epigenetic enzymes, such as epigenetic writers, remodelers, readers, and in particular epigenetic erasers such as ten-eleven translocation (TET) enzymes have been associated to NAFLD.^21^ In diet-induced NAFLD mice, Kim *et al*. showed that changes in the gut microbiome after a dietary change correlated to persistent modifications in liver DNA methylation, suggesting the gut microbiome may play a part in altering hepatic DNA methylation in NAFLD.^22^ However, there is a lack of studies on the interaction between the gut microbiome and hepatic DNA methylation in humans.

Recently, we reported a trend toward improvement of the histological NASH necro-inflammation score upon vegan allogenic FMT in individuals with NAFLD compared to autologous FMT.^23^ In the current analysis, we employed a multi-omics machine learning approach to investigate possible mechanisms behind the observed trend. We hypothesized that FMT, by changing the gut microbiota composition, can alter plasma levels of gut-derived metabolites. Moreover, FMT could impact liver DNA methylation, either directly or by way of an altered metabolite influx from the gut. To our knowledge, the effect of FMT on liver DNA methylation has not been studied in humans to date. Here, we describe the effects of FMT on gut microbiota composition and plasma metabolomics signature, after which we analyze the liver DNA methylation changes upon vegan allogenic or autologous FMT. Lastly, we use multi-omics correlation analyses to investigate the relations between FMT-induced changes in gut microbiota, plasma metabolites, and liver DNA methylation.

## Results

Data from 21 treatment-naïve participants with metabolic syndrome and hepatic steatosis on ultrasound, treated with either allogenic (*n* = 10) or autologous (*n* = 11) FMT are included in these analyses. Participants with a history of cardiovascular disease, T2DM, renal disease, cholecystectomy or compromised immunity were excluded. Included participants did not use any medication. The complete inclusion and exclusion criteria are given elsewhere.^23^ The baseline characteristics of study participants are given in Table 1. Importantly, there was no significant difference in age, and baseline dietary intake was similar between the treatment groups (Supplementary Table S1). With regard to NAFLD severity at baseline (i.e. percentage of steatosis, NAFLD activity score (NAS) and fibrosis stage), there were no significant differences between the groups (Table 1).

**Table 1.** Baseline characteristics of 21 individuals with biopsy-proven NAFLD. Data is presented as mean ± standard deviation, median [interquartile range], or count (percentage). p-values represent results of t-test for normally distributed data, Mann-Whitney U tests for non-normally distributed data, and Fisher’s exact tests for categorical data.

		Autologous FMT (*n* = 11)	Allogenic FMT (*n* = 10)	*p*-value
Age, years		48.5 ± 10.2	51.2 ± 6.6	0.48
Sex, male/female		10/1	7/3	0.31
BMI, kg/m^2^		31.5 ± 4.8	31.7 ± 3.5	0.91
HbA1c, mmol/mol		37.6 ± 3.8	38.2 ± 3.7	0.70
Glucose, mmol/L		5.7 ± 0.5	5.8 ± 0.7	0.79
AST, IU/L		29.0 [26.5–33.0]	39.5 [37.0–49.5]	**0.001**
ALT, IU/L		48.1 ± 16.5	70.8 ± 23.4	**0.02**
ALP, IU/L		83.0 [54.0–120.5]	71.0 [58.8–76.8]	0.67
GGT, IU/L		41.1 ± 21.4	45.1 ± 19.3	0.66
Cholesterol, mmol/L		5.8 ± 1.6	6.0 ± 0.8	0.75
HDL-C, mmol/L		1.2 [1.0–1.4]	1.2 [1.0–1.4]	0.80
LDL-C, mmol/L		4.0 ± 1.3	4.2 ± 0.7	0.71
Triglycerides, mmol/L		1.2 ± 0.6	1.4 ± 0.5	0.41
CRP, mg/mL		2.2 [0.8–4.3]	1.5 [0.9–3.2]	0.50
Steatosis, %		35.0 ± 20.7	34.1 ± 20.4	0.92
NAS score	1	1 (9%)	0 (0.0%)	0.38
	2	5 (46%)	4 (40%)	
	3	4 (36%)	2 (20%)	
	4	1 (9%)	4 (40%)	
Necro-inflammation score	0	1 (9%)	0 (0%)	0.06
	1	10 (91%)	6 (60%)	
	2	0 (0%)	4 (40%)	
Fibrosis stage	F0	3 (30%)	2 (20%)	1.00
	F1	6 (60%)	5 (50%)	
	F2	2 (20%)	2 (20%)	
	F3	0 (0%)	1 (10%)	

data are expressed as mean ± SD or median [interquartile range], depending on the distribution of the data. ALP: Alkaline phosphatase; ALT: Alanine transaminase; AST: Aspartate transaminase; BMI: Body mass index; CRP: C-reactive protein; FMT: Faecal microbiota transplantation; GGT: Gamma-glutamyltransferase; Hba1c: Glycated hemoglobin; HDL-C: High-density lipoprotein cholesterol; LDL-C: Low-density lipoprotein cholesterol; NAS score: NAFLD activity score; T2DM: Type 2 diabetes mellitus.

Our ML model was able to accurately discriminate allogenic from autologous FMT recipients based on changes in gut microbiota (AUC 0.78), plasma metabolomics (AUC 0.74) and liver DNA methylation profiles (AUC 0.75) between time points 0 and 24 weeks (Supplementary Figures S1–3). Permutation analysis showed that the likelihood that the obtained accuracies were due to chance was very low (0.88; *p* < 0.001). The strongest discriminative features between the groups in each analysis are described in the following sections.

### Gut microbial composition changes upon allogenic versus autologous FMT

**Figure 1.**
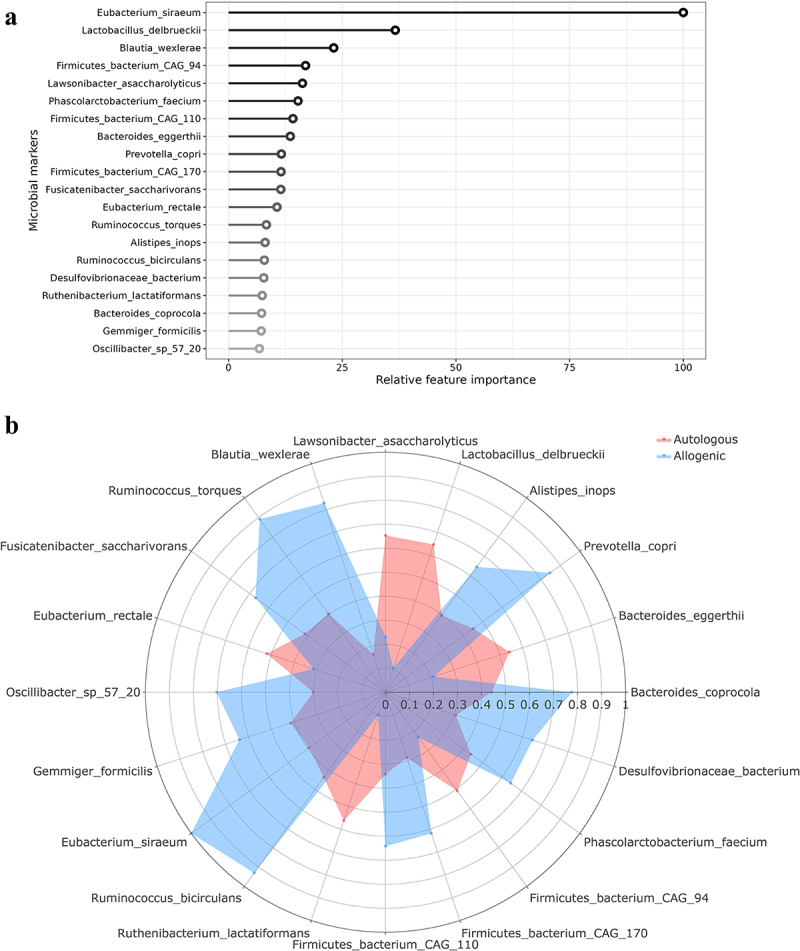
Changes in gut microbiota after vegan allogenic or autologous FMT. (a) Top 20 most discriminative gut microbial strains found by the machine learning model. The most important feature is set to 100% with the other features relative to the most important feature; (b) Spider plot of relative delta differences in the top 20 microbes between the vegan allogenic and autologous FMT groups. The values are rescaled between 0.1 and 1.

Allogenic and autologous FMT had differential effects on the gut microbiota composition of recipients. Paired samples for metagenomics shotgun microbiota data before and after FMT were available for 17 participants. The top 20 most discriminative fecal microbes between the two FMT groups are given in Figure 1a. *Eubacterium siraeum* and *Blautia wexlerae* were increased upon allogenic FMT, whereas their abundance was unchanged or decreased in most participants upon autologous FMT. Contrastingly, *Lactobacillus delbrueckii* decreased in most participants upon allogenic FMT, whereas it was unchanged or increased upon autologous FMT. See Figure 1b for relative differences per bacteria upon FMT.

### Plasma metabolite changes upon allogenic versus autologous FMT

**Figure 2.**
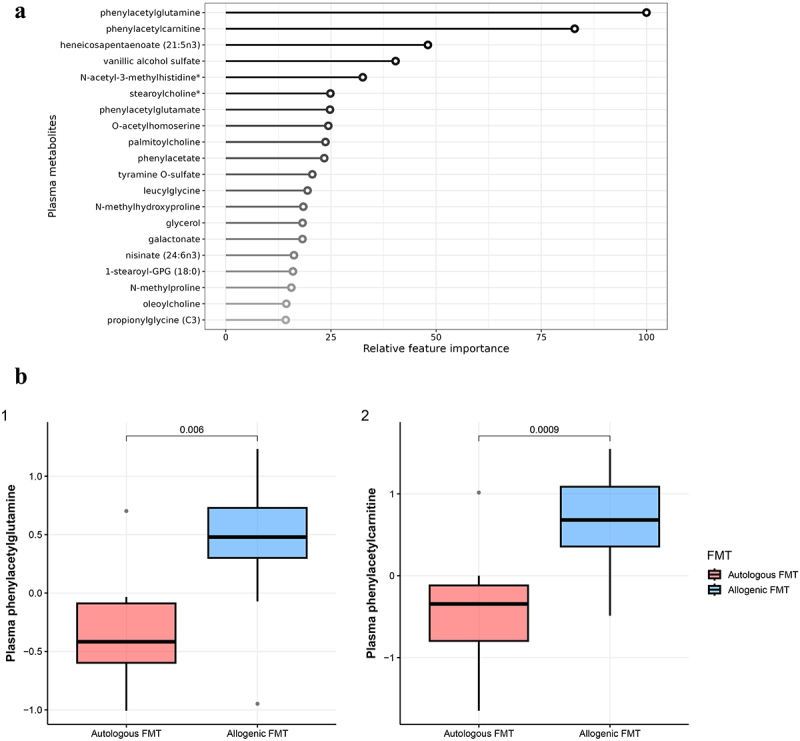
Changes in plasma metabolites after vegan allogenic or autologous FMT. (a) Top 20 most discriminative plasma metabolites found by the machine learning model. The most important feature is set to 100% with the other features relative to the most important feature; B (1): Boxplot of phenylacetylglutamine B (2): Boxplot of phenylacetylcarnitine.

Next, we show that fasting plasma metabolite profiles of recipients were distinctly altered upon allogenic versus autologous FMT. Paired plasma metabolomics data was available for all 21 participants before and after FMT. Figure 2a depicts the top 20 most discriminative metabolites. The two most discriminative metabolites between the FMT groups were phenylacetylcarnitine and phenylacetylglutamine; both are involved in phenylacetate metabolism and significantly increased upon allogenic FMT whereas they decreased upon autologous FMT (Figure 2b). Moreover, several metabolites related to glycerolipid metabolism were identified as most discriminative between allogenic and autologous FMT: glycerol; 1-stearoyl-GPG (18:0); palmitoyl-arachidonoyl-glycerol (16:0/20:4); 1-palmitoyl-GPG (16:0). Three choline-derived metabolites were among the top 20 most discriminative metabolites between the groups, i.e. stearoylcholine, palmitoylcholine and oleoylcholine. Plasma levels of these metabolites increased after autologous FMT, whereas they remained largely unchanged in allogenic FMT recipients.

### Liver DNA methylation changes upon autologous versus allogenic FMT

**Figure 3.**
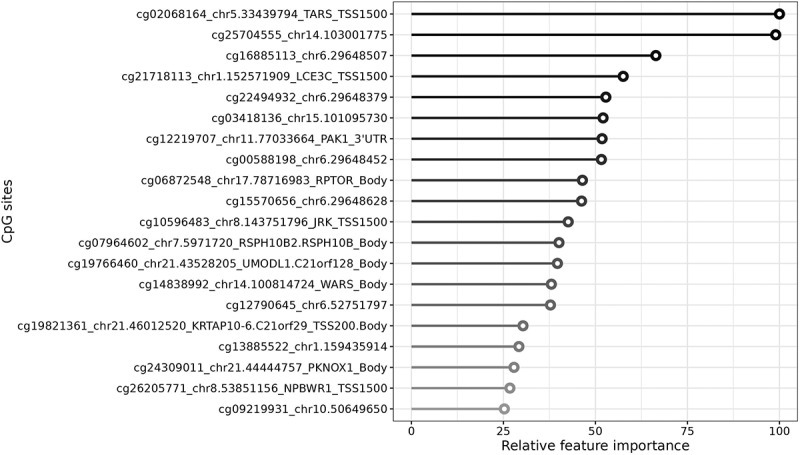
Top 20 most discriminative methylation changes in CpG sites in the liver after vegan allogenic or autologous FMT found by the machine learning model. The most important feature is set to 100% with the other features relative to the most important feature.

Paired liver DNA methylation profiles before and after FMT were available for 20 participants. The top 20 differentially methylated CpGs (dmCpGs) that together were most important for distinction between treatment groups are shown in Figure 3. The top-ranked dmCpG was cg02068164 within the transcription start site 1500 (TSS1500) of Threonyl-TRNA Synthetase 1 (*TARS)* which decreased in methylation upon allogenic FMT.

Within Late Cornified Envelope 3C *(LCE3C*) cg21718113, in the TSS1500, fourth in relative importance methylation also decreased upon allogenic FMT. Moreover, there was a trend toward hypermethylation in the allogenic versus hypomethylation in the autologous FMT group of cg06872548 in Regulatory Associated Protein Of MTOR Complex 1 (*RPTOR)*, on chromosome 17.

Multiple loci within Zinc finger protein 57 (*ZFP57)* on chromosome 6 were in the top 20 dmCpGs, showing decreased in methylation upon allogenic FMT (Supplementary Figure S4a, b).

### Multi-omics correlations

**Figure 4.**
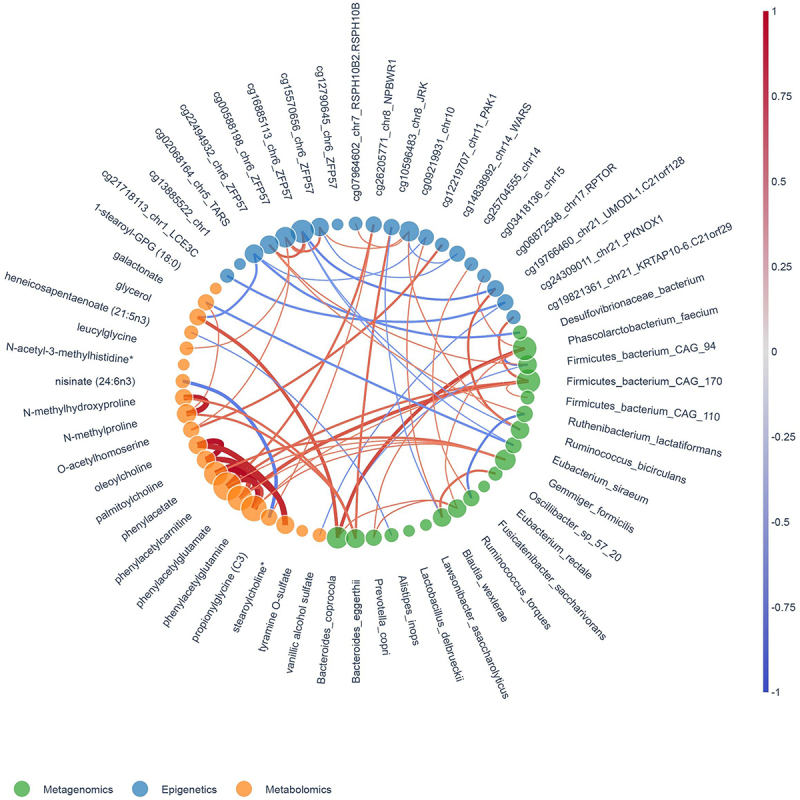
Multi-omics correlations of differentially altered features between vegan allogenic and autologous FMT. The top 20 most discriminatives gut microbial strain (green nodes), plasma metabolites (orange nodes) and differentially methylated CpGs (blue nodes) are displayed. The size of the nodes represents the number of correlations. Line thickness and colour depicts direction and strength of correlation (blue: positive correlation; red: inverse correlation), based on Spearman correlation coefficients (ρ).

Finally, we analyzed multi-omics correlations between liver DNA methylation, plasma metabolites and fecal microbiota before and after FMT. A spearman correlation (cutoff <−0.6/>0.6) was applied to integrate the top 20 features from each omics set (DNA methylomics, metagenomics and metabolomics) and find multi-omics interactions upon FMT. The correlation plot depicted in Figure 4 shows which features were correlated with each other; the line thickness represents the strength of the association, and the color represents the direction of the correlation (between red for positive and blue for negative). We found that *E. siraeum*, the most discriminative feature from the metagenomics analysis, was negatively correlated with cg16885113 in *ZFP57*. As for plasma metabolites, it was notable that the phenylacetate metabolites (phenylacetate, phenylacetylcarnitine, phenylacetylglutamate, and phenylacetylglutamine) all correlated positively with the intestinal bacterial species *Firmicutes bacterium CAG 170* and *Gemminger formicilis*, suggesting these microbes may function as phenylalanine converters. Not surprisingly, there were also strong correlations between these metabolites themselves.

The most important feature from the liver DNA methylation analysis, cg02068164 within the *TARS* gene, was negatively associated with the metabolite glycerol. As for microbe-epigenetic correlations, *Blautia wexlerae* was positively associated with DNA methylation at cg12219707, in the 3’UTR of the P21 (RAC1) Activated Kinase 1 (*PAK1)* gene and negatively correlated with cg10596483 annotated to the Jrk Helix-Turn-Helix Protein (*JRK)* gene. This last dmCpG was also positively correlated with abundance of *Bacteroides eggerthi.*

When studying the relation of the identified most discriminative dmCpGs with liver histology data of the participants, we found a correlation between cg16885113 in *ZFP57* and hepatocyte ballooning at baseline (Supplementary Figure S5).

## Discussion

Using a multi-omics approach, we have deeply characterized the changes that occur in the microbiome, metabolome, and – uniquely – liver DNA methylome, upon allogenic vegan donor versus autologous FMT in individuals with NAFLD. We have identified correlations between liver DNA methylation, gut microbes, metabolites, and histological features of NAFLD.

ML models can be used to address highly complex biological datasets and have previously in epigenetic studies.^24^ Here, we used ML to distinguish between the two intervention groups. It is important to note that the combination of all variables in each multi-omic dataset is used to make this distinction, and therefore specific variables may not be strongly related to either group when considered individually. To identify relevant changes upon FMT, we looked into the top 20 most discriminative features from each ML model. We identified several variables that may be of interest in microbiota-mediated effects on the liver of individuals with NAFLD.

### Vegan FMT increases abundance of Blautia wexlerae, a potential anti-obesogenic probiotic

Among the top differentially altered microbes, *E. siraeum* and *B. wexlerae* both increased in abundance after vegan allogenic FMT. *E. siraeum* has previously been linked to NAFLD, and in non-human primates its abundance was positively correlated with high-density lipoprotein cholesterol levels.^25,26^ In our study, we observed an inverse correlation between changes in *E. siraeum* abundance and methylation of cg16885113 in *ZFP57*, further discussed below. *B. wexlerae* is a strictly anaerobic bacterium that has potential probiotic properties, including the production of bactericins that inhibit the colonization of pathogenic bacteria.^27^ *Blautia* species, especially *B. wexlerae* and *B. luti*, are depleted in the gut microbiota of children with obesity.^28^ *Blautia* abundance has been found to increase in individuals with NAFLD who started a hypocaloric high protein diet.^29^ A recent Japanese cross-sectional cohort study showed that the abundance of *B. wexlerae* was inversely correlated with obesity and T2DM. The authors subsequently showed that oral administration of *B. wexlerae* to mice on a high-fat diet led to decreased body weight and improved insulin sensitivity.^30^ Our findings indicate that *B. wexlerae* abundance increases after vegan donor FMT and it may thus be a transferable microbe from a vegan diet.

### Vegan FMT increases hepatic production of phenylacetate metabolites

Plasma levels of the metabolites phenylacetylglutamine (PAG) and phenylacetylcarnitine (PAC) increased in study participants after receiving allogenic FMT as compared to autologous FMT. The presence of the gut bacteria *Gemmiger formicillis* and *Firmicutes bacterium*_*CAG*_170 was positively associated with these two metabolites. Both PAG and PAC are produced in the liver during the degradation of phenylacetic acid, which is derived from microbial catabolism of phenylalanine in the gut.^31^ The role of phenylacetic acid in inducing mitochondrial dysfunction and hepatocyte lipid accumulation is currently under investigation.^32^ PAG has been suggested to promote cardiovascular disease via signaling in adrenergic receptors, yet urinary PAG has been positively correlated with microbial gene richness in individuals with obesity.^33,34^ A recent study employing a network pharmacology approach identified PAG as a distinctive feature of NAFLD, suggesting it may be a biomarker for hepatic dysfunction.^35^ PAC is involved in fatty acid transport into the mitochondria.^36^ It has been shown that conversion of mitochondrial acetylcarnitine to acetyl-CoA in the nucleus provides a source of acetyl groups for histone acetylation.^37^ Whether PAC is involved in these epigenetic processes requires further investigation.

### Choline-derived metabolites are increased after autologous FMT

Our analyses revealed differential changes in plasma levels of three long-chain acylcholines (i.e. stearoylcholine, palmitoylcholine and oleoylcholine) between the autologous and allogenic FMT recipients. Acylcholines are products of choline metabolism, which primarily takes place in the liver. Choline is an essential nutrient that is mostly obtained through diet, and plasma levels of choline-derived metabolites are influenced by gut microbial composition.^38,39^ Importantly, it is known that choline deficiency contributes to nonalcoholic fatty liver disease, as phosphatidylcholine plays a critical role in the assembly of very low-density lipoprotein (VLDL) particles that are essential in transporting fat and cholesterol from the liver.^40,41^ Another choline metabolite, trimethylamine N-oxide (TMAO), is a widely known harmful microbial product associated with an increased risk of cardiovascular disease.^38,42^ Interestingly, choline acts as a methyl donor, and some studies have shown that choline availability has a large impact on DNA methylation.^38^ However, we did not identify correlations between these acylcholines and specific CpG sites in the liver in our multi-omics analysis. Our data support previous studies that suggest a link between microbial composition and the production of acylcholines.^41,43,44^

Our findings that plasma levels of microbial-derived metabolites (i.e. phenylacetate and choline metabolites) are differentially altered between participants receiving allogenic versus autologous FMT indicate that FMT can induce metaorganismal pathway changes from the gut bacteria to the liver.

Furthermore, several plasma metabolites related to glycerolipid metabolism were differentially altered between allogenic and autologous FMT receivers. Glycerol is released from white adipose tissue as a result of lipolysis, and its flux to the liver initiates hepatic gluconeogenesis.^45^ In NAFLD, adipose tissue insulin resistance increases lipolysis and the glycerol flux to the liver, increasing hepatic gluconeogenesis.^46^ Alterations in glycerol levels and its related metabolites after FMT may thus be indicative of changes in these metabolic processes.

### FMT affects hepatic DNA methylation

Our analyses identified multiple dmCpGs in the liver following allogenic or autologous FMT. The top feature was hypermethylation of cg02068164 in *TARS* in the autologous group, while it was hypomethylated in the allogenic group. TARS is a protein that is essential for gene translation, as it catalyses the covalent binding of threonine to tRNA during the process of adding amino acids to the polypeptide chain.^47^ Although *TARS* is not tissue specific, it has been shown to be expressed in the liver.^48^ *RPTOR* was another gene in which we identified dmCpGs. *RPTOR* encodes mTOR, a regulator of liver autophagy in multiple liver diseases, including NAFLD.^49^ Notably, multiple dmCpGs in *ZFP57* were identified among the top most important features. ZFP57 belongs to the KRAB zinc finger proteins group and is a regulator of the epigenetic process of imprinting.^50^ Mutations in *ZFP57* are associated with an imprinting disorder in which DNA methylation is altered, causing transient neonatal diabetes.^51^ This phenomenon of multiple CpGs within the same gene has not been seen in previous studies. While it is yet unclear how these changes will affect disease progression, these findings suggest that *ZFP57* may act as a hepatic regulator in response to gut microbiota-derived signals.

### Strengths and limitations

To our knowledge, this is the first study to investigate the impact of FMT on liver DNA methylation in humans, in the context of NAFLD or any other liver condition. With this work, we demonstrate that multivariate omics models can be utilized to identify relevant CpG sites that are differentially methylated and to correlate multi-omics changes between FMT groups in participants with NAFLD. A key strength of this study is the use of liver biopsy specimens for DNA methylation analysis, eliminating the need to use surrogate tissue (such as commonly used peripheral blood mononuclear cells) to infer epigenetic changes. Moreover, because the FMT groups were well matched in age and participants were all treatment-naïve, we were able to analyze samples without age- or medication bias, two factors known to affect both gut microbiota composition and epigenetic marks. Furthermore, other potential confounders, such as dietary intake or NAFLD severity, were well matched between groups. However, it is important to note that the sample sizes were small, and therefore this study should be viewed as a conceptual work. Devoted clinical trials are necessary to confirm the associations noted in this study. Since paired samples from each participant before and after the intervention were compared, we did not have to account for genetic (*cis*) associations within the analyses. As the field of epigenetics is quickly expanding, there are several aspects that we did not address in this work. While our study investigated overall DNA methylation profiles in the liver, it would be highly interesting to explore alterations in single-cell DNA methylation in the liver to identify specific cell types involved in the epigenetic changes described here. Moreover, differentiating methylation from de-methylation could provide further insights into the epigenetic changes that occur after FMT. Additionally, it should be noted that DNA methylation is only one of the epigenetic tools that can affect the transcription machinery, and that some genes may still be transcribed despite being in a methylated state.^52^ Exploring the cross-talk between different epigenetic markers and investigating mitochondrial DNA methylation could also provide further insights into the epigenetic mechanisms at play. Finally, while our analyses specifically focused on gut bacteria, other components of the microbiome such as bacteriophages may also influence metabolic processes after FMT.

### Conclusions

Manipulation of the gut microbiome through FMT can alter plasma levels of microbial metabolites, such as phenylacetate- and choline-derived metabolites, as well as liver DNA methylation in individuals with NAFLD. Distinct multi-omics relations exist between gut microbiota, plasma metabolites and liver DNA methylation. This lends support to further therapeutic exploration of the gut-liver axis in treatment development for NAFLD.

## Material and methods

This is a post-hoc analysis of liver DNA methylation, fecal metagenomics and plasma metabolomics alterations in a single-center, double-blind, randomized controlled study in which the effect on histologically assessed NAFLD of three 8-weekly lean vegan donor (allogenic; *n* = 10) FMTs was compared to own (autologous; *n* = 11) FMTs using paired liver biopsies (trial registration no.: NL4189-NTR4339).^23^ This study was approved by the Amsterdam Medical Centers ethics committee (AMC METC 2013_207). All participants provided written, informed consent.

Liver biopsies were performed for the purpose of this research, as this is currently the reference standard for the diagnosis of NAFLD.^53^ The age-matched recipients of allogenic or autologous FMTs were Caucasian, overweight (BMI >25 kg/m^2^), treatment – naïve and omnivorous individuals with hepatic steatosis determined by ultrasound. Study participants were asked to record their food intake for 7 days before the first FMT visit. Liver biopsies, fasting plasma, and fecal samples were collected at baseline and at 24 weeks, and stored for analyses as previously described.^23^ A schematic overview of the number of participants included in each analysis is shown in Table 1.

### Faecal metagenomics

Fecal samples were collected at baseline and at 24 weeks after start of treatment. Gut microbial DNA was isolated from fecal samples using the Maxwell® 16 Instrument (Promega, Leiden, The Netherlands). Microbial DNA was analyzed for microbiome composition by shotgun metagenomics sequencing. Raw reads were checked and quality-filtered using fastp (v.0.20.0).^54^ Here, the adapter was detected and removed, 5 bp in front for read1 was trimmed, and sliding window quality trimming was applied (with a window width of 4 bp and threshold Q-score of 15). After trimming and adapter removal, reads shorter than 70 bp were removed. Paired-end reads that passed the quality filtering were then mapped against the human genome (hg19) using Bowtie 2 (v.2.4.1).^55^ The settings include very-sensitive and inclusion of dovetail, where mates extend past each other. SAMtools (v.1.9) was used to convert SAM to BAM and to remove the reads that were mapped to the human genome. Sambamba (v.0.7.1) was used hereafter to sort the unmapped reads by name with a memory limit of 40 gigabytes. BEDtools (v.2.27.1) was used to convert the sorted unmapped reads to forward and reversed fastq format reads.^56–58^ The remaining high‐quality, non-human reads were subsampled to 20 million paired‐end reads per sample using seqtk (v.1.3r106). The forward and reversed reads are concatenated and fed to the HUMAnN3 pipeline (v.3.0.0.alpha.3).^59^ For each sample, species‐level microbial composition with viruses added in relative abundance was inferred using MetaPhlAn3 (v.3.0.2).^59^ After mapping the reads against the pangenomes selected based on inferred composition (using Bowtie 2), unmapped reads were translated and mapped against the full UniRef90 protein database using DIAMOND (v.0.9.32).^60^ MetaCyc pathway community‐level abundance was normalized to copies per million (CPM).

### Plasma metabolomics

Fasting EDTA plasma samples were taken at both time points and analyzed by METABOLON (Morrisville, NC, USA) for ultra-high-performance liquid chromatography coupled to tandem mass spectrometry (LC-MS/MS) untargeted metabolomics, as previously described.^61^ To minimize the effect of artifacts in the downstream analysis, metabolomics intensities underwent heavy curation by pre-filtering, including missing data imputation, and normalization of data values. This was performed using a Perseus platform. Originally, METABOLON measured 1299 metabolites, and after removing unknown metabolites, 1022 metabolites underwent imputation and normalization for further analysis. Per metabolite, the median value was scaled to one and samples below detection threshold were imputed with the lowest measured value. Filtering for xenobiotics resulted in 805 metabolites for Machine Learning (ML) modeling. Differential analysis was conducted with two methods: ANOVA and Kruskal Wallis.

### Liver DNA isolation and DNA methylation profile

DNA was isolated from liver biopsies taken at baseline and 24 weeks, see Supplementary Methods for details. DNA methylation profiles were generated using the Illumina Infinium MethylationEPIC BeadChip 850k array. Quality control and pre-processing of DNA methylation data was performed in R Studio (v3.5.1) using the Bioconductor (v3.7) packages minfi (v1.26.2) for import, functional normalization for normalization, MethylAid (v1.14.0) and shinyMethyl (v1.16.0) for quality control.^62,63^ Samples were included for further analysis if they passed default MethylAid parameter thresholds and were not outliers according to principal component analysis. Probes were excluded from the analyses if they were suspected to be promiscuous.^64^ Since both male and female participants were present in the cohort, all probes annotated to allosomes were removed. Furthermore, probes were excluded if their gene body or CpG of interest overlapped with a known SNP with a minor allele frequency >1% per the included Illumina manifest annotation file, or when probes were supposed to include unknown genetic variation detected through implementation of the gaphunter function (threshold 20%, >2 clusters) available under minfi.^65^

### Machine learning

We applied a classification algorithm to identify which parameters (delta values as relative changes between point 0 and 24 weeks) best predicted allocation of treatment groups, that is, autologous or allogenic FMT.^66^ For each -omics modality (microbes, metabolites, and CpGs), a model was deployed. Features were filtered prior to each simulation on the different -omics modality to reduce dimensionality. First, an unsupervised variance threshold was applied, removing features which show low variation, independent on the treatment group. For microbes, metabolites and CpGs, a variance threshold of 0.01, 0.25, and 0.027 was utilized, respectively. Hereafter, a univariate feature selection was applied (35%, 20%, and 1% for microbes, metabolites and CpGs, respectively), resulting in 41, 49, and 82 features for the microbial, metabolic, and CpG modality, respectively.

Within each ML simulation, the models were constructed with the same stability selection procedure to ensure robust results and prevent overfitting.^67^ For this, we reshuffled the order of the samples in the original dataset 100 times. After each shuffle, the dataset was split up in a training- and testing dataset, with the division of 80/20. Within the training dataset, a three-fold cross-validation was applied to tune the hyper parameters of the model and to improve accuracy and control for overfitting. The number of trees used was 2000. Performance of the different models was estimated via an area under the curve (AUC) of the test dataset to distinguish allogenic FMT receivers from the autologous FMT control group. The final performance metric is a mean AUC with standard deviation and a mean feature importance over the different shuffles. The ML pipeline was implemented in python v 3.7.7, using the scikit-learn (v 0.23.1) package.^68^

### Statistical analyses

T-tests or Mann-Whitney *U* tests were performed to detect differences in baseline characteristics, depending on normality of the data distribution. Differences in distribution of categorical parameters between the groups at baseline were tested by Fisher’s exact tests. Boxplots distinguishing the autologous- from allogenic FMT group were analyzed using the Mann-Whitney *U* test. Correlations between DNA methylation loci and clinical scores (e.g., steatosis grade, ballooning, fibrosis grade, NAS score and inflammation score) and between the different -omics modalities were analyzed using the Spearman rank correlation. A *p*-value below 0.05 was considered statistically significant. The statistical analyses were performed using R version 4.0.2

## Supplementary Material

Supplemental Material

## Data Availability

The raw DNA methylation data and metagenomic sequencing data generated for this study have been published under controlled access for research purposes at the European Genome-phenome Archive at EGAS00001006893, https://ega-archive.org.
