# Supplementary figures and images for: Faecal Microbiota transplantation affects liver DNA methylation in Non-alcoholic fatty liver disease: a multi-omics approach
Source: Gut Microbes. 2023 Jun 14;15(1):2223330. doi: 10.1080/19490976.2023.2223330 (PMC10269428; doi:10.1080/19490976.2023.2223330)

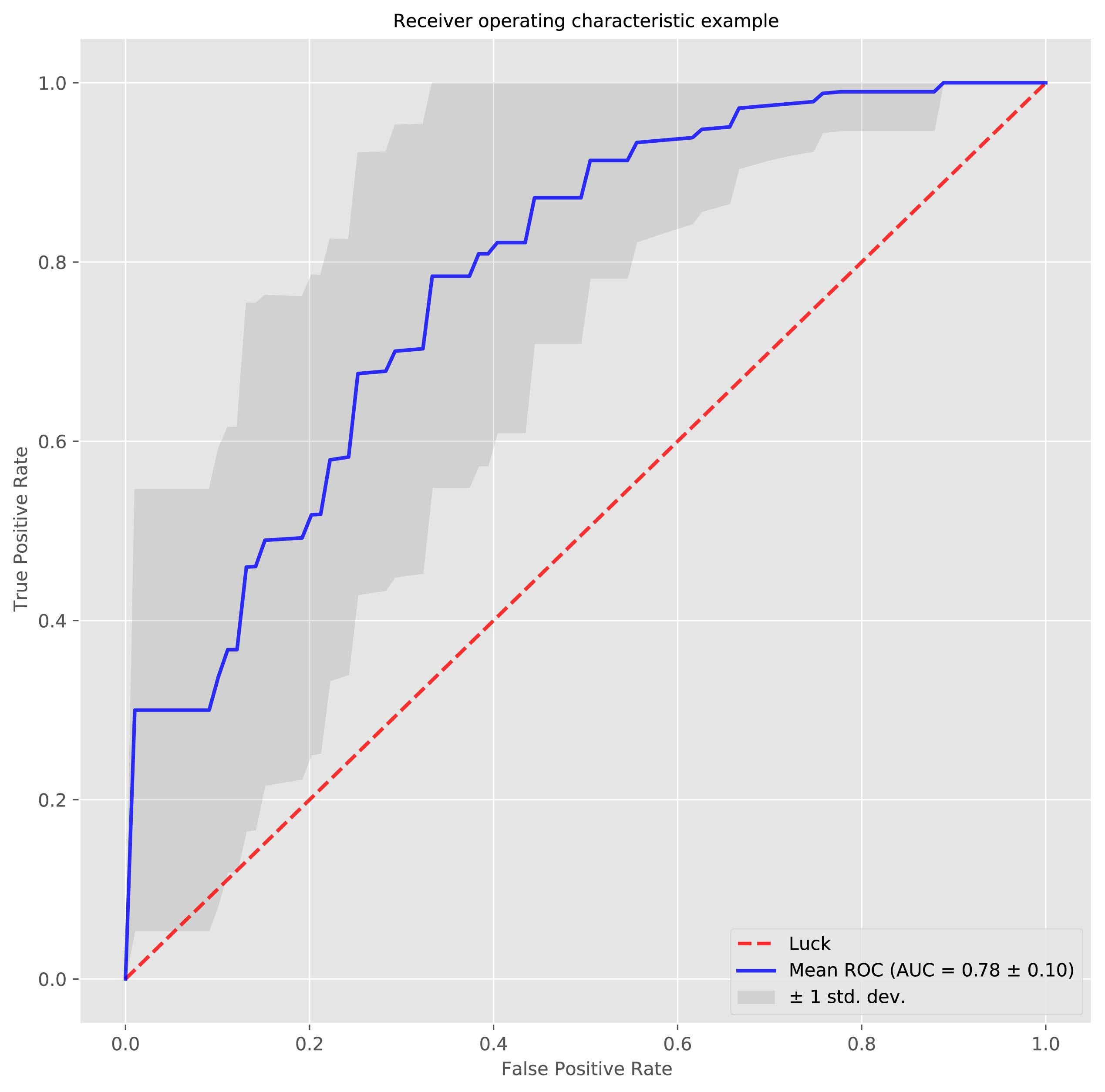

Supplement: Supplemental Material [file KGMI_A_2223330_SM4401.zip › Supplementary material/Suppl Fig 1__AUC microbiota.jpg]

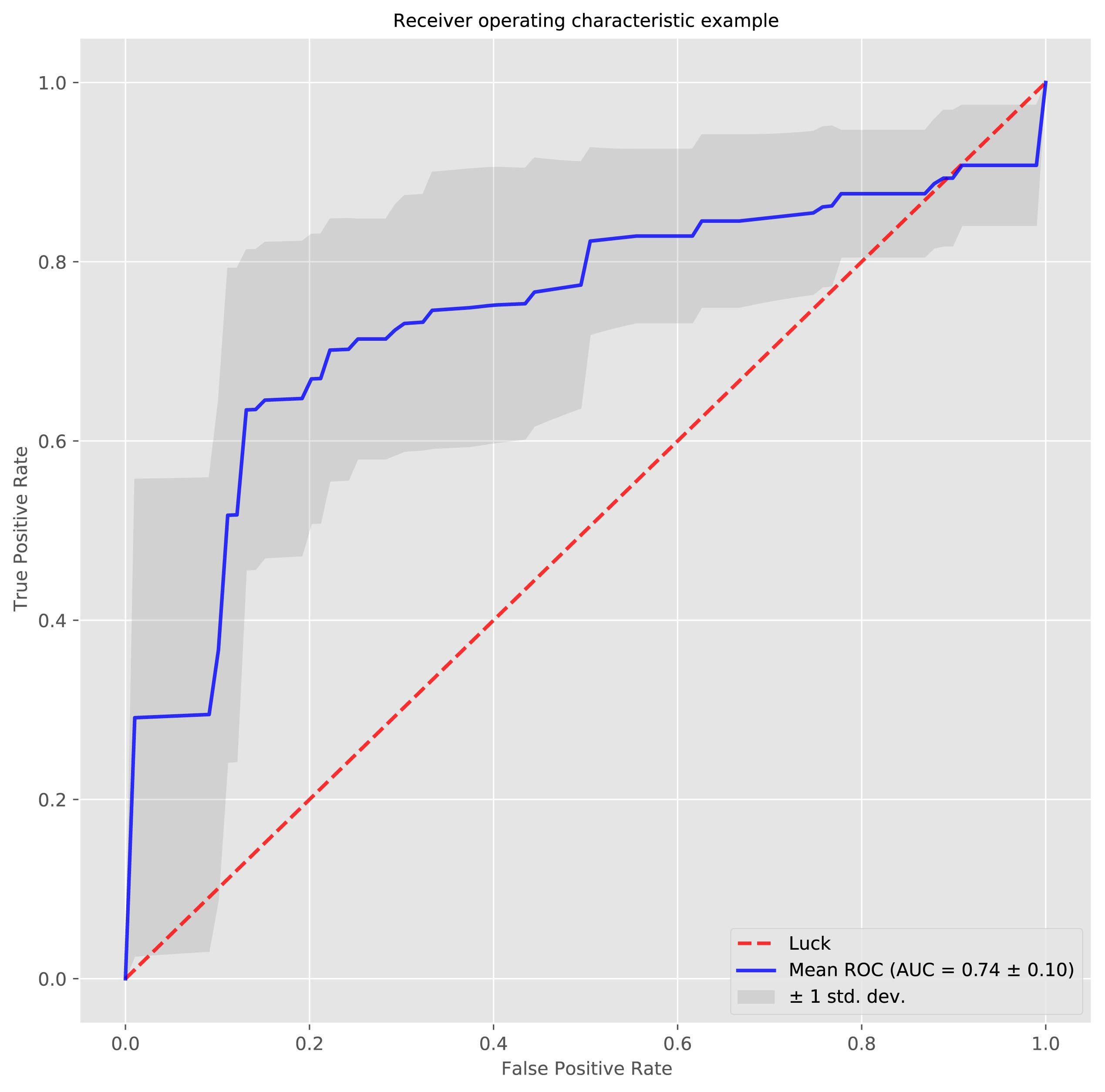

Supplement: Supplemental Material [file KGMI_A_2223330_SM4401.zip › Supplementary material/Suppl Fig 2_AUC plasma metabolomics.jpg]

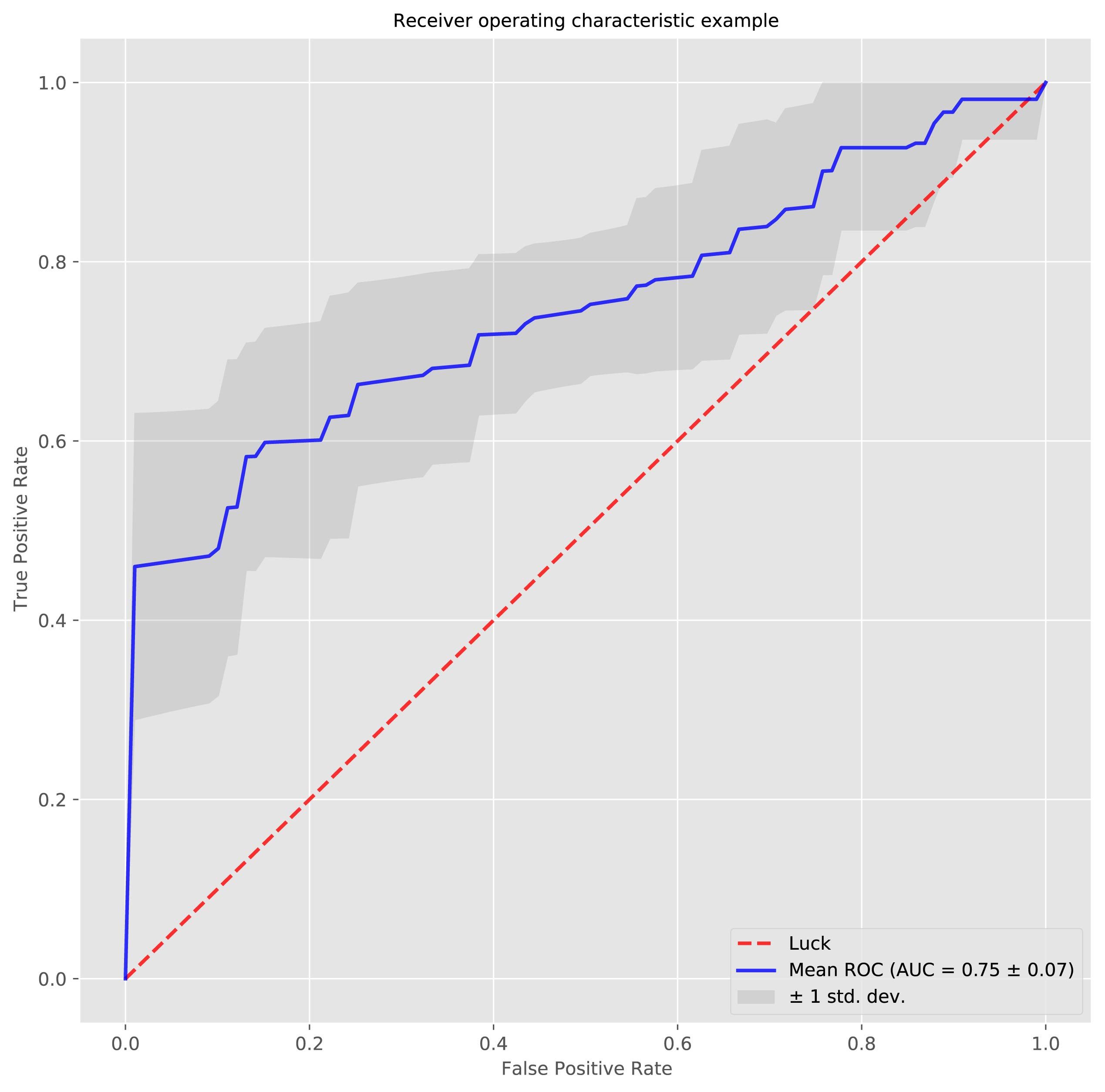

Supplement: Supplemental Material [file KGMI_A_2223330_SM4401.zip › Supplementary material/Suppl Fig 3_AUC DNA methylation.jpg]

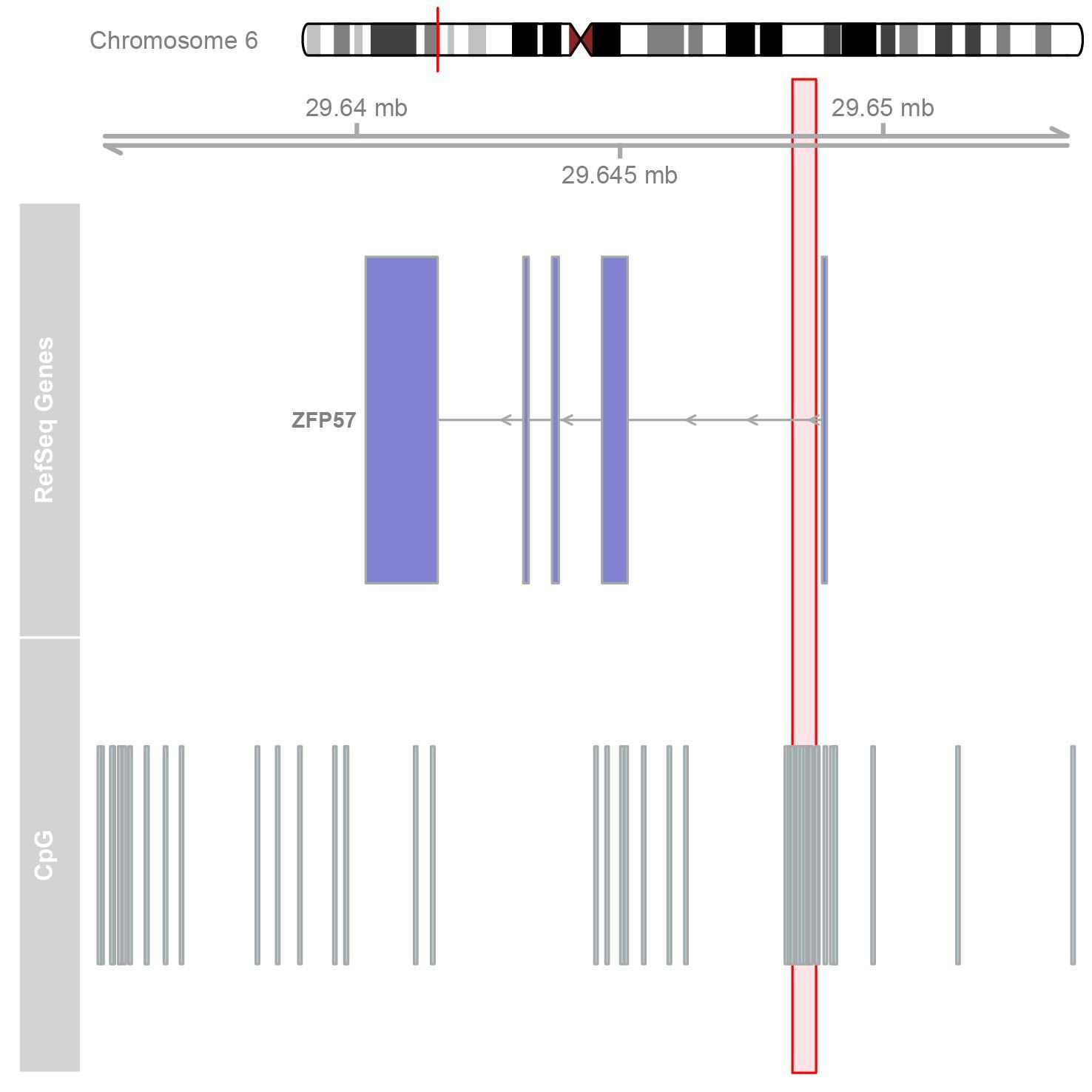

Supplement: Supplemental Material [file KGMI_A_2223330_SM4401.zip › Supplementary material/Suppl Fig 4A_revised_Location of dmCpGs in Ch6_ZFP57.jpg]

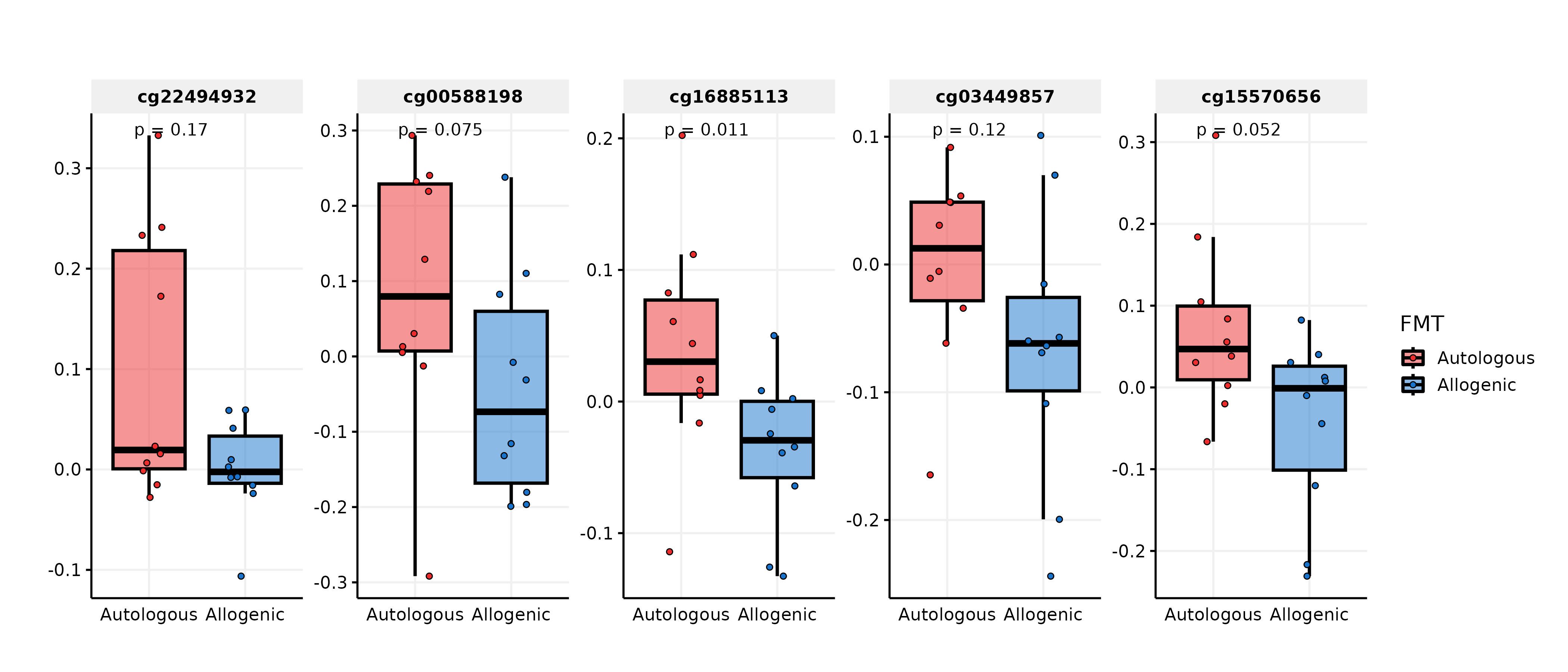

Supplement: Supplemental Material [file KGMI_A_2223330_SM4401.zip › Supplementary material/Suppl Fig 4B_Boxplots of dmCpGs of ZFP57.jpg]

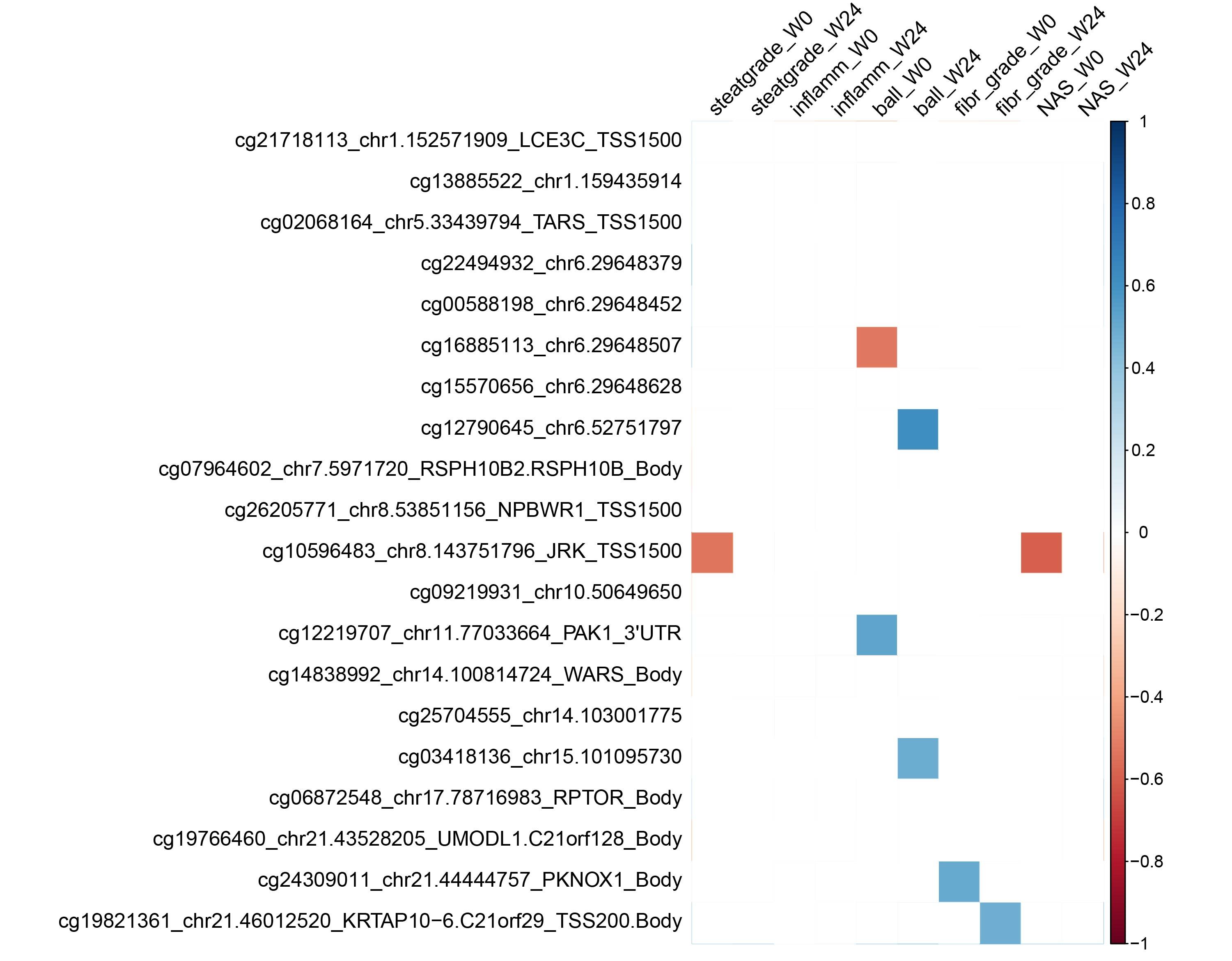

Supplement: Supplemental Material [file KGMI_A_2223330_SM4401.zip › Supplementary material/Suppl fig 5_heatmapcprrelations dmCpGs and liver histology.jpg]
